# Low oxygen tension increases mitochondrial membrane potential and enhances expression of antioxidant genes and implantation protein of mouse blastocyst cultured in vitro

**DOI:** 10.1186/s13048-017-0344-1

**Published:** 2017-07-20

**Authors:** Yun-Yi Ma, Huei-Wen Chen, Chii-Ruey Tzeng

**Affiliations:** 1 Center for Reproductive Medicine, Department of Obstetrics and Gynecology, Taipei Medical University Hospital, Wuxing Street 252, Taipei, Taiwan; 2 Graduate Institute of Toxicology, College of Medicine, National Taiwan University, Taipei, Taiwan; 3 Department of Obstetrics and Gynecology, School of Medicine, College of Medicine, Taipei Medical University, Taipei, Taiwan; 4 Graduate Institute of Clinical Medicine, Taipei Medical University, Taipei, Taiwan

**Keywords:** Antioxidant, Hypoxia, Implantation, Mitochondria, Mouse embryo

## Abstract

**Background:**

In human IVF, embryos cultured with a lower O_2_ tension (5%) can give rise to higher success rates when compared with normoxic conditions (20%). However, the mechanisms behind the beneficial effects of reduced oxygen tension in embryogenesis remain unclear. The aim of this study was to evaluate the expression of oxygen related and antioxidant genes and mitochondrial function in mouse embryo cultured under hypoxic and normoxic conditions, to investigate the beneficial effect of low oxygen tension in preimplantation embryogenesis.

**Methods:**

Two-cell ICR mouse embryos were cultured to blastocysts under different oxygen tension (3% and 20%). The gene expression of oxygen-related proteins (hypoxia-inducible factor, HIF), HIF targets (vascular endothelial growth factor, VEGF; glucose transporter 3, GLUT-3) and antioxidants (manganese superoxide dismutase, MnSOD; peroxiredoxin 5, PRDX5) were assessed using quantitative RT-PCR and implantation-related protein (Leukemia Inhibitory Factor Receptor, LIFR) was validated by immunofluorescence. Apoptosis, mitochondrial membrane potential (MtMP) and ROS levels were measured by TUNEL, JC-1 and DCFDA assays, respectively.

**Results:**

Blastocyst development rate (92.3% vs. 79.4%) and hatch rate (80% vs. 70.4%) were both higher in embryos cultured in 3% O_2_ than in 20% O_2_. The transcription levels of MnSOD, PRDX5, VEGF and GLUT-3 also significantly increased in 3% O_2_ compared with 20% O_2_ (*P* < 0.05). Immunofluorescence showed that the intensity of staining for HIF-2α, MnSOD and LIFR were higher in 3% O_2_. Blastocysts cultured under 3% O_2_ exhibited significantly higher MtMP compared with 20% O_2_. ROS and Apoptosis levels were significantly higher in the 20% O_2_ group than in the 3% O_2_ group (*P* < 0.05).

**Conclusions:**

Low O_2_ tension may improve embryo viability by increasing expression of antioxidant enzymes and glucose transporter activities. It provides an environment conducive to viability by upregulation of LIFR/VEGF and increased MtMP which could enhance implantation potential and reduce apoptosis in mouse blastocyst. These effects may be initiated and regulated by HIF-2α, a key mediator in a hypoxic environment.

## Background

Most mammals have a lower concentration of O_2_ inside the uterus than in the oviducts [1]. The O_2_ concentration in the oviduct of rhesus monkeys has been found to range from 5% to 8.7%, while in the uterus it was only about 1.5 to 2% or 2 to 8% in mammals [2]. Therefore, the embryos are exposed to reduced O_2_ tension at the time they arrive in the uterus, which corresponds to the time of blastocyst formation and implantation. Therefore we chose 3% O_2_ concentration in our study to investigate the beneficial effect of low oxygen tension in preimplantation embryogenesis.

Embryo culture systems are a vital component of assisted reproductive technology for the production of animal or human embryos, and many factors affect the development of mammalian preimplantation embryos in vitro. It is well known that environmental oxygen (O_2_) affects embryo development and the intracellular redox balance [3]. Numerous studies in various species have shown that embryo development can be significantly improved by culturing embryos under low O_2_ tension [4, 5]. Similarly, recent preliminary clinical studies have shown a positive effect of hypoxia in human IVF and ICSI procedures that used a lower O_2_ tension (5%) during days 3–5 of in vitro culture, which gives higher cleavage, implantation, pregnancy, and birth rates in humans [3, 6–9] compared with conventional culture in an atmosphere of 20% O_2_. These findings provide evidence that low O_2_ tension is more efficient and generates a higher number of and better quality embryos than atmospheric O_2_.

Low O_2_ tension in the oviduct and uterus is thought to influence subsequent competence for embryo development [10]. Many studies suggest that low O_2_ tension triggers a wide range of cellular events centered on the regulation of hypoxia-inducible factors (HIFs), which may play important roles in these events [11]. The beneficial effects and mechanism of action of HIFs in reduced O_2_ tension during embryogenesis remain unclear.

HIFs are recognized as the master transcriptional regulators of cellular hypoxic responses, and consist of two basic helix–loop–helix protein subunits (HIFα and HIFβ); the α subunit is only stable in cells under low O_2_ conditions [12, 13]. HIF1α, HIF2α, HIF3α, and HIF1β (also known as aryl hydrocarbon nuclear translocator or ARNT) have been reported to be constitutively expressed. Activation of HIFα and HIFβ (which form a heterodimer) by hypoxia is regulated in an O_2_-dependent fashion at the level of the stability of the α-subunit protein [14]. At atmospheric O_2_ tension, HIFα protein is rapidly degraded because of O_2_-dependent hydroxylation by prolyl hydroxylase domain-containing proteins and a von Hippel–Lindau tumor suppressor protein-dependent ubiquitin–proteasome pathway [15].

The effects of HIF are primarily mediated by a hypoxic transcriptional response through activation of the expression of a large number of genes involved in glycolysis, angiogenesis, proliferation, erythropoiesis, and hematopoiesis [16–18]. Therefore, HIF is required for embryonic survival in reduced O_2_ environments [19]. Moreover, some studies using HIFα-knockout mouse models have demonstrated embryonic lethality involving poor fetal survival and severe vascular defects [20, 21].

During development from the 2-cell stage to the blastocyst stage, the embryo gradually increases its mitochondrial membrane potential, which relates to mitochondrial activity, to maintain rapid embryo growth and metabolism. At the same time, levels of reactive O_2_ species (ROS) are increased [22]. ROS, a by-product of aerobic respiration and metabolism, are a result of a redox imbalance, and cause DNA damage and induce apoptosis, lipid peroxidation, and oxidative modification of proteins [23]. The redox balance of cells is regulated by several antioxidant enzymes, including superoxide dismutase (SOD), peroxiredoxin (PRDX), and glutathione (GSH) [24]. A number of studies report that the detrimental effect of oxidative stress on embryos under atmospheric O_2_ concentrations is correlated with increased generation of ROS [25–27].

Therefore by using RT-qPCR and immunofluorescence staining, the objective of this study was to examine whether O_2_ tension affects embryo development, HIF (HIF-1α and HIF-2α) gene expression profiles and the target gene of HIFs including, vascular endothelial growth factor (VEGF), a key of angiogenesis and implantation [28], glucose transporter 3 (GLUT-3), the glucose uptake gene which regulates metabolism and energy supply of mouse embryo [29] and antioxidant enzymes (MnSOD and PRDX5) which play important role in anti-apoptosis and survival of blastocyst. The levels of apoptosis and mitochondrial activity in mouse embryos cultured under hypoxic (3% O_2_) and normoxic (20% O_2_) conditions during 2-cell to blastocyst formation were evaluated using TUNEL and JC-1 staining, respectively. We sought to determine whether low O_2_ tension can influence embryonic developmental competence, antioxidant capacity, and implantation potential during the preimplantation period through activation of the genes involved in these processes.

## Methods

### Embryo collection and culture

This study was approved by the Animal Care and Use Committee of Taipei Medical University (Taipei, Taiwan). Imprinting control region (ICR) mice (6–8 weeks old) were superovulated with 10 IU of pregnant-mare serum globulin (PMSG) (Sigma-Aldrich, St. Louis, MO, USA), which was followed 48 h later by 10 IU human chorionic gonadotropin (HCG) (Sigma-Aldrich). 2-cell stage Embryos (E1.5) were collected from oviducts and ovaries 66 h after HCG treatment. About 30–50 Embryos were cultured per drop in human tubal fluid (HTF) medium (Irvine Scientific, Santa Ana, CA, USA) supplemented with 10% serum substitute (Irvine Scientific) and randomly assigned to either low (3%) or high (20%) oxygen tensions in a 5% CO_2_, 37 °C in each experiment. The same type of incubator (Astec, APM-30D, Japan) was used for embryo culture. Embryo morphology and development were monitored daily until day 4 of the blastocyst stage (E3.5 ~ 4.5). Blastocyst development and hatch rates were assessed on daily basis by calculated proportion of embryos developed to the blastocyst stage (Blastocoel cavity more than half the volume of the embryo) and hatching stage (trophectoderm has started to protrude through the zona) [30] after cultured 48–72 h. Four replicate experiment were performed.

### RNA extraction

Total RNA was extracted from pools of 50–65 expanded blastocysts from per treatment, using Tri Reagent (Sigma-Aldrich) according to the manufacturer’s instructions. The frozen blastocysts were thawed, vortexed for 20 s, and allowed to sit at room temperature for 5 min. Chloroform (Sigma-Aldrich) was added, samples were vigorously shaken, and then incubated at room temperature for 15 min. Samples were centrifuged at 13,000 rpm for 15 min at 4 °C and the upper aqueous phase was removed. Isopropanol (Sigma-Aldrich) was added and RNA was precipitated by overnight incubation at −80 °C, followed by centrifugation at 13,000 rpm for 30 min at 4 °C. RNA pellets were washed with 70% ethanol, air-dried, and redissolved in 10 mL of sterile water. The RNA solution was kept frozen at −80 °C until all samples were assembled for reverse transcription.

### Amplified reverse transcription and gene expression analysis

The reverse transcription assay was carried out using a QuantiTect Whole Transcriptome Kit (207,043; Qiagen, Hilden, Germany). The gene expression levels of (i) HIFs: HIF-1α and HIF-2α, (ii) HIF target genes: GLUT-3 and VEGF, and (iii) antioxidant enzyme genes: MnSOD and PRDX5 were analyzed by quantitative real-time PCR (RT-qPCR) performed in an Applied Biosystems 7300 Real-Time PCR System (Applied Biosystems) with SensiMix SYBR ® Hi-ROX kit (Bioline, Reagents Ltd., USA). The procedure was as follows: 95 °C for 10 min; 40 cycles of 95 °C for 10 s and 60 °C for 30s; melting curve from 65 to 95 °C. A cycle threshold (Ct) was calculated for each sample using the GeneAmp 7300 software. The Primer sequences are listed in Table 1. The comparative Ct method was used for quantification of mRNA expression levels using the amplification efficiency of each gene. All results were normalized to a reference gene (β-actin) and expressed as fold change from the 20% O_2_ group.

**Table 1:** List of the primer sequences used for real-time RT-PCR experiment

Gene	Primer Sequence (5′–3′)
*HIF-1α*	Forward:ACCTTCATCGGAAACTCCAAAG Reverse:ACTGTTAGGCTCAGGTGAACT
*HIF-2α*	Forward:TAAAGCGGCAGCTGGAGTAT Reverse:ACTGGGAGGCATAGCACTGT
*GLUT3*	Forward:ATGGGGACAACGAAGGTGAC Reverse:GTCTCAGGTGCATTGATGACTC
*VEGF*	Forward:CTGCCGTCCGATTGAGACC Reverse:CCCCTCCTTGTACCACTGTC
*MnSOD*	Forward:GCACATTAACGCGCAGATCA Reverse:AGCCTCCAGCAACTCTCCTT
*PRDX5*	Forward:CGGAAAGAAGCAGGTTGGGA Reverse:CATCTGGCTCCACGTTCAGT
*β-actin*	Forward:ACGGCCAGGTCATCACTATTG Reverse:CAAGAAGGAAGGCTGGAAAAGA

*HIF-1α* hypoxia-inducible factors 1α, *HIF-2α* hypoxia-inducible factors 2α, *GLUT3* glucose transporter 3, *VEGF* vascular endothelial growth factor, *MnSOD* manganese superoxide dismutase, *PRDX5* peroxiredoxin 5

### Immunofluorescence and confocal microscopy

Blastocysts were fixed in 3.7% paraformaldehyde in PBS for 20 min at room temperature and washed twice in PBS containing BSA (BPBS). Embryos were permeabilized with 0.2% Triton X-100 in PBS for 30 min, then washed twice in BPBS. Embryos were incubated in blocking solution (0.1% Tween 20 in BPBS) for 1 h and then incubated with primary antibodies against HIF-2α (Abcam, USA; ab199) (1:100), MnSOD (Abcam; ab16956) (1:200), and LIFR (Santa Cruz, USA; sc-659) (1:200) overnight at 4 °C. On the following morning, embryos were rinsed three times in BPBS and incubated in the appropriate secondary antibody conjugated with Alexa 488–labeled goat antimouse IgG (Invitrogen; A-11029) (1:100) or Alexa 568–labeled goat antimouse IgG (Invitrogen; A-11036) (1:100) for 1 h in the dark. After washing in PBS, the stained embryos were mounted in Fluoroshield mounting medium with 4′,6-diamidino-2-phenylindole (DAPI; Abcam, ab104139) and observed by confocal laser-scanning microscopy (FV1000; Olympus, Japan) to detect the fluorescence. For image analysis, the intensities of green fluorescence (Alexa 488) and red fluorescence (Alexa 568) were measured using Olympus Fluoview® software (FV10-ASW).

### Measurement of ROS and Mitochondrial Membrane Potential (MtMP)

2′,7′-Dichlorofluorescein diacetate (DCHFDA; Sigma-Aldrich) was used to quantify H_2_O_2_ generation in blastocysts by measuring the intensity of fluorescence. Blastocysts from each treatment group were incubated in HTF supplemented with 10 μM DCHFDA for 15 min at 37 °C. After incubation, blastocysts were washed three times with PBS containing 1 mg/mL polyvinyl pyrrolidone (PVP-PBS), mounted onto glass slides and visualized immediately under an epifluorescence microscope at 490 nm excitation and 525 nm emission.

MtMP in blastocysts was measured by staining with 5,5′,6,6′-tetrachloro-1,1′,3,3′-tetraethylbenzimidazolylcarbocyanide iodide (JC-1) (Life Technologies, Rockville, MD, USA). Embryos were incubated in HTF containing 5.0 mg/mL JC-1 for 15 min at 37 °C in the dark, washed with PBS, then observed at either 510 nm (green mitochondria/J-monomer) or 590 nm (red-to-orange mitochondria/J-aggregate) using a confocal microscope (TCS SP5; Leica, Germany). Acquired images were analyzed using Image J® software (1.47v), which allowed for quantitation of the fluorescence signal intensity of the DCHFDA- or JC-1-stained embryos. The ratio of red (J-aggregate) to green (J-monomer) staining was determined for all sections of the embryo, from which an average ratio of J-aggregate to J-monomer staining for the entire embryo was determined. Both experiments were replicated three times with a group of 10–20 blastocysts in each replicate.

### Terminal Deoxynucleotidyl Transferase-mediated dUTP Nick-end Labeling (TUNEL) assay

Blastocysts were washed three times in PVP-PBS followed by fixation in 3.7% paraformaldehyde in PBS for 1 h. After fixation, the embryos were washed in PVP-PBS and permeabilized by incubation in 0.5% Triton X-100 for 1 h. The embryos were then washed twice in PVP-PBS and incubated with fluorescein-conjugated dUTP and terminal deoxynucleotidyl transferase (In Situ Cell Death Detection Kit, Roche Molecular Biochemicals) in the dark for 1 h at 37 °C. After counterstaining incubated embryos with 25 mL Hoechst 33,258 (Sigma-Aldrich) for 20 min in the dark to label all nuclei, embryos were washed in PVP-PBS, mounted with slight coverslip compression, and examined using a confocal laser scanning microscope (FV1000; Olympus).

### Statistical analysis

Differences in ratios of embryo development were analyzed by Chi-square tests. After log transformed, Kolmogorov-Smirnov test was performed for normality test. The comparison of JC-1 staining, ROS and TUNEL assays were analyzed by student’s t-test. Differences in gene expression were analyzed by student’s t-test after log transformed. Data were expressed as mean ± SE. All analyses were performed using the SAS statistical package (ver. 9.3 for Windows; SAS Institute, Cary, NC, USA), and two-tailed *P* values <0.05 was assumed statistically significant.

## Results

### Blastocyst development and hatch rates

The results of four replicate experiments including a total of 185 2-cell embryos cultured in 3% O_2_ and 189 cultured in 20% O_2_ showed that embryos cultured in 3% O_2_ had significantly higher rates of blastocyst development (92.3% vs. 79.4%) and hatching (80% vs. 70.4%) (*P* < 0.05; Table 2) compared with those cultured in 20% O_2_.

**Table 2:** Both blastocyst development and hatch rates were higher in embryos cultured in 3% O_2_ than in 20% O_2_

Oxygen concentration	3% O_2_	20% O_2_	*P* value*
No. 2-cell embryo	185	189	
No. Blastocyst(%)	167(92.3)	150(79.4)	0.0033
No. Hatching blastocyst(%)	148(80)	133(70.4)	0.0074

*Chi-square test

### RT-qPCR

**Fig. 1:**
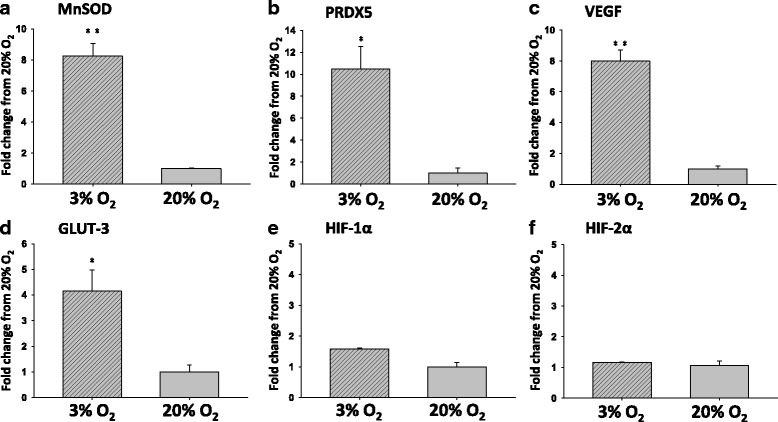
The transcription levels of antioxidant enzymes (MnSOD and PRDX5) and HIF target genes (VEGF and GLUT-3) significantly increased in 3% O_2_ than 20% O_2_ . Results detected by RT-qPCR were mean ± SE calculated by the ΔΔCt method, normalized to β-actin. Means with different superscripts were significantly different (*P < 0.05, **P < 0.0001)

Expression of MnSOD and VEGF was significantly higher (*P* < 0.0001; Fig. 1a, c), and that of PRDX5 and GLUT-3 was also higher (*P* < 0.05; Fig. 1b, d) in the 3% O_2_ group. The levels of expression of GLUT-3 and VEGF were 4.15- and 7.99-fold higher, respectively, in the 3% O_2_ group compared with the 20% O_2_ group (*P* < 0.05). Analysis of expression of antioxidant genes in blastocysts showed that the transcription levels of MnSOD and PRDX5 genes were 8–10-fold higher in the 3% O_2_ group compared with the 20% O_2_ group.

### Immunofluorescence staining

The presence of all proteins examined was determined by immunolocalization using specific antibodies. All epifluorescence data were detected using the same microscope settings, and evaluated by visual assessment of the intensity observed in merged images.

**Fig. 2:**
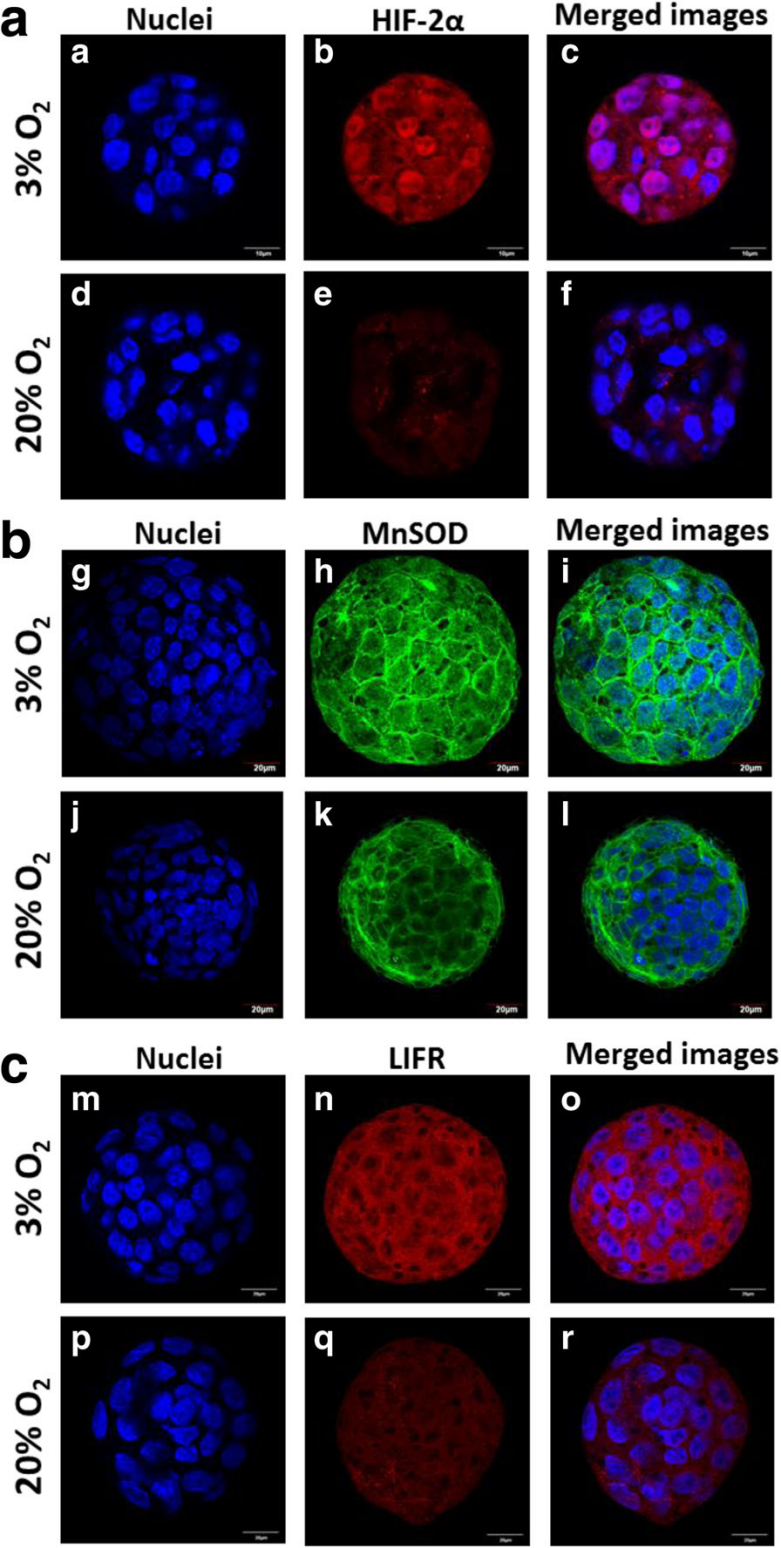
Immunofluorescence staining images showed that **a** HIF-2α, **b** MnSOD and **c** LIFR protein expression levels were all higher in embryos cultured in 3% O_2_ than in those cultured in 20% O_2_. Secondary antibody conjugated with Alexa flour 568-labeled HIF-2α (b, e), LIFR (n, q), and Alexa 488-labeled flour MnSOD (h, k) proteins with DAPI staining of nuclei (a, d, g, j, m, p) and merged images (c, f, i, l, o, r). Scale bar 10 or 20 μm

Immunofluorescence staining showed that the intensity of staining for HIF-2α, MnSOD, and LIFR was higher in the 3% O_2_ group compared with the 20% O_2_ group (Fig. 2), although no significant difference was seen in the levels of HIF-2α mRNA. HIF-2α was observed to be predominantly localized to the nucleus in blastocysts cultured in 3% O_2_. In contrast, blastocysts cultured in 20% O_2_ showed reduced HIF-2α staining intensity (Fig. 2a).

### ROS, TUNEL assays, and JC-1 staining

**Fig. 3:**
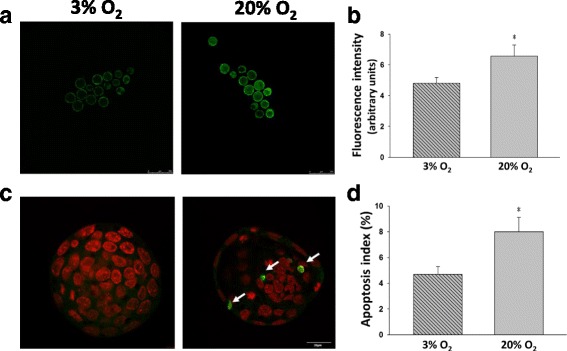
Both ROS **a**, **b** and apoptosis **c**, **d** levels were significantly higher in 20% O_2_ than 3% O_2._ **a** Representative images of intracellular ROS levels in 3% and 20% O_2_ cultured blastocysts after staining with DCHFDA. **b** Fluorescent intensity of intracellular ROS levels were higher in 20% O_2_ (*n* = 29) than in 3% O_2_ (*n* = 31). **c** Apoptotic nuclei (long arrow) identified by TUNEL were increased in mouse blastocysts cultured under 20% O_2_. Pseudocolor was representation of nuclei with DAPI. **d** The apoptotic index was higher in blastocysts cultured in 20% O_2_ (*n* = 35) than in 3% O_2_ (*n* = 31), expressed as the percentage of apoptotic nuclei divided by the total number of nuclei in each blastocyst. Scale bars 250 and 20 μm. (*P < 0.05)

Fluorescent intensity of ROS levels were significantly higher (*P* < 0.05) in the 20% O_2_ group (6.57 ± 0.71) than in the 3% O_2_ group (4.81 ± 0.37) (Fig. 3b). In addition, the apoptosis index showed that the blastocysts from the 20% O_2_ group had a significantly higher (*P* < 0.05) percentage of apoptotic cells (8.0 ± 1.1%) compared with those from the 3% O_2_ group (4.7 ± 0.6%) (Fig. 3d).

**Fig. 4:**
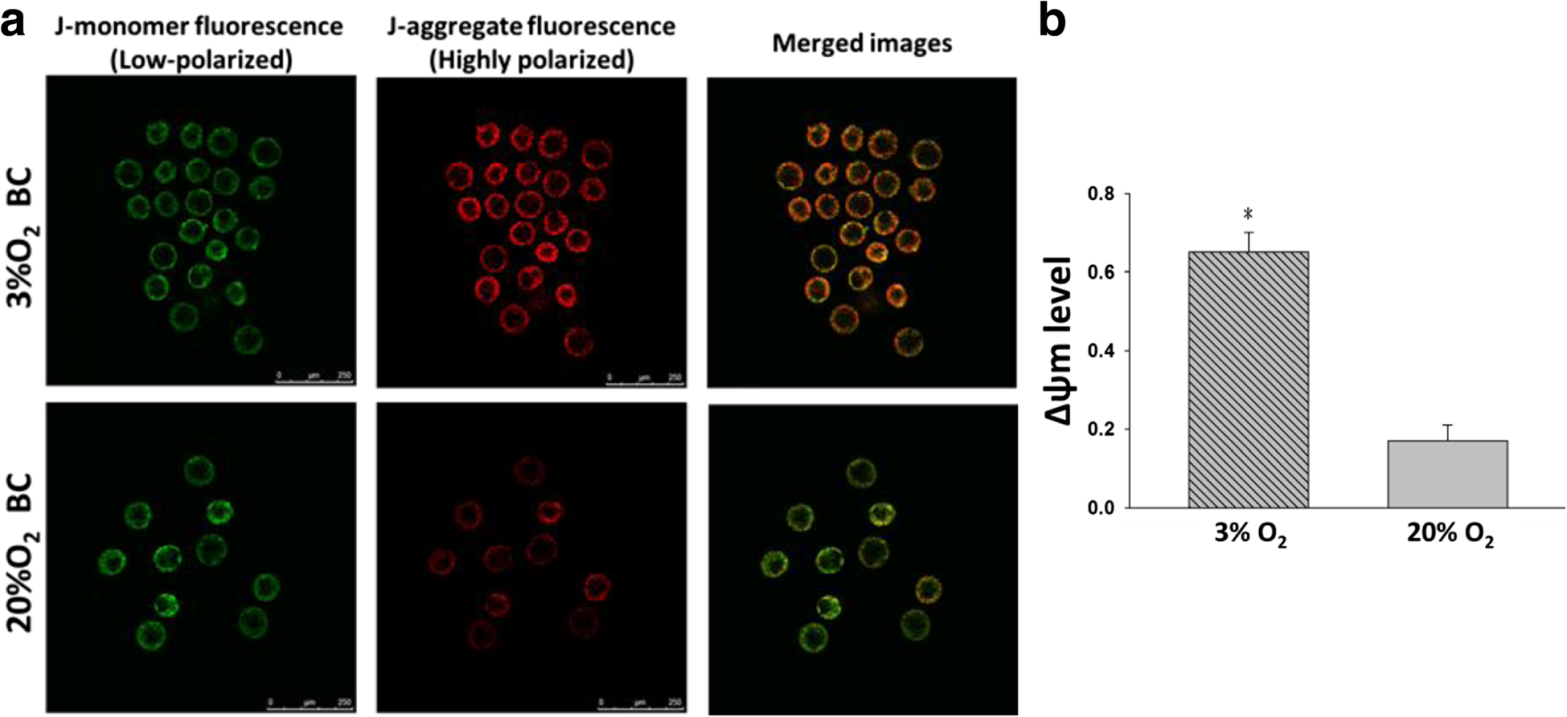
Blastocysts cultured under 3% O_2_ exhibited significantly higher mitochondrial membrane potential than 20% O_2_. **a** Mitochondrial membrane potential (ΔΨm) visualized by JC-1-staining in blastocysts cultured under 3% and 20% O_2_, respectively. **b** The level of ΔΨm was significantly higher in blastocysts cultured in 3% O_2_ (*n* = 22) compared with those cultured in 20% O_2_ (*n* = 20) (*P < 0.05). Scale bar 250 μm. The level of ΔΨm was computed by obtaining the ratio of J-aggregate to J-monomer immunofluorescence intensity for each individual blastocyst section

JC-1 staining and TUNEL assays were performed in blastocysts generated from 2-cell cultures under 3 and 20% O_2_. After JC-1 staining, blastocysts fluoresced green at 488 nm, red at 543 nm is seen (Fig. 4a). The ratio of red and green fluorescence (ΔΨm) was significantly higher in blastocysts cultured in 3% O_2_ (0.65 ± 0.05) compared with those cultured in 20% O_2_ (0.17 ± 0.04) (*P* < 0.001) (Fig. 4b).

## Discussion

Many studies suggest that lower O_2_ benefits the development of embryos from the 8-cell to blastocyst stage, which is consistent with studies showing low O_2_ tension in the uterus. Aside from follicular O_2_ tension, in humans several factors in the follicular microenvironment also potentially influence the oocyte competence and consequently the embryo competence [31]. The in vivo O_2_ concentration in the oviduct and uterine environment is lower than the atmospheric O_2_ (20%) typically used for the culture of somatic cells. Time-lapse monitoring [32, 33] of developmental arrest in human and mouse preimplantation embryos cultured in atmospheric O_2_ tension has demonstrated that embryo development is delayed under atmospheric O_2_ tension compared with low O_2_ tension [34]. Our previous study demonstrated that the expression of hemeoxygenase-1 (OH-1), a known HIF target gene, was upregulated during blastocyst hatching [35]. The current study indicated that culturing mouse embryos in low O_2_ tension (3%) during the preimplantation period can not only increase the rate of 2-cell to blastocyst development (*P* < 0.05), but can also promote the development of embryos as evidenced by a higher hatching rate (*P* < 0.05) that is similar to that of embryos in vivo. These results are consistent with similar mouse [36], bovine [37], and human [38] studies. By contrast, the reduction in blastocyst numbers seen under 20% O_2_ is likely because of the effects of oxidative stress on the embryos associated with increased generation of ROS-induced DNA damage in cells. Some of this variation may relate to the strains of mice used, or to differences in culture media composition.

We found that HIF-1α mRNA expression was slightly increased by low O_2_ concentration, and that the expression of HIF-2α was higher and predominantly localized to the nucleus in blastocysts cultured under 3% O_2_ compared with those cultured under 20% O_2_. HIF-2α shares 48% amino sequence identity with HIF-1α and therefore shares common functional domains and can activate genes encoding a hypoxia response element (HRE) [39]. However, many knockout studies demonstrate that HIF-1α and HIF-2α have distinct expression patterns and functions [40, 41].

These results are consistent with those of a previous study [42] that found that the transcription and synthesis of both HIF-1α and HIF-2α were constitutive and seemed not to be affected by O_2_. The O_2_-dependent regulation of HIF-1α and HIF-2α may be mediated by the same mechanisms. Some reports indicate that HIF-2α is relatively resistant to O_2_-dependent degradation [43], while HIF-1α is tightly regulated at the protein level through ubiquitin-mediated degradation under normoxic conditions, meaning that it is rapidly degraded by O_2_ and has a short half-life [44]. This might explain their distinct expression patterns and function. Therefore, we hypothesized that HIF-2α may be a major regulating modulator in hypoxia.

Oxidative stress in in vitro culture is detrimental to embryos, causing retardation or arrest in development [45]. In mammals, there are two main protections against ROS during embryo development. External protection takes place in follicular and tubal fluids, and involves many nonenzymatic antioxidants. Internal protection is mediated by antioxidant enzymes such as SOD and peroxiredoxin, which are encoded by transcripts in oocytes and embryos [46]. Addition of antioxidants to culture media produces better results and overcomes blockages in development; it also reduces the O_2_ concentration [47, 48]. In this study, we evaluated the protective capacity of hypoxic conditions for embryos.

PRDX5, one of the six PRDX isoforms (PRDX1 to PRDX6) in mitochondria can improve blastocyst development by protecting cells from oxidative stress by regulation of ROS/reactive nitrogen species (RNS) homeostasis [49, 50]. MnSOD, an endogenous mitochondrial antioxidant enzyme, can maintain mitochondrial ROS homeostasis through the transformation of toxic superoxides (O^−^_2_), by-products of the electron transport chain, into hydrogen peroxide (H_2_O_2_) [51, 52]. Blastocysts cultured in low O_2_ had higher levels of MnSOD (*P* < 0.0001) and PRDX5 (*P* < 0.01) transcription than did blastocysts cultured in high O_2_. These results are consistent with those of a study in cattle [53], suggesting that low O_2_ conditions can enhance the antioxidant-mediated defense against oxidative stress by scavenging the extracellular ROS generated, thereby providing embryos with a low oxidative stress environment. As expected, MnSOD was also higher in blastocysts cultured under 3% O_2_. These data provide mounting evidence that internal protection in embryos is activated by hypoxia through increased production of these antioxidant enzymes to limit ROS during subsequent development. Knockout animal studies revealed that MnSOD is a target of HIF-2α and is responsible for the maintenance of ROS as well as for mitochondrial homeostasis [54, 55]. Immunofluorescence studies of HIF-2α suggested that, under hypoxic conditions, MnSOD expression may be regulated by HIF-2α through modulating ROS levels in embryos. The increase in MnSOD levels in blastocysts cultured in 3% O_2_ corresponds with reductions in both ROS and the apoptosis index in our blastocysts.

Many genes are known to contain the HIF binding site HRE, including glycolytic enzymes (GLUT-3) and angiogenesis factors (VEGF) [18]. At the cellular level, the response to hypoxia includes a switch from anaerobic metabolism to anaerobic glycolysis, where the glucose transporters facilitate glucose uptake across the plasma membrane. GLUT-3 is present on the apical membrane of the trophectoderm cells in mouse blastocysts and is believed to mediate the uptake of glucose into the blastocyst from the external environment [19, 56]. The expression of GLUT-3 was upregulated in the 3% O_2_ group compared with the 20% O_2_ group (*P* < 0.05), which may increase the glucose uptake to generate sufficient amounts of ATP without producing excessive amounts of ROS, thereby leading to an elevation of the membrane potential in mitochondria. This upregulation of expression was similar to that observed by Kind et al. [57], and is consistent with other reports that human [58] and mouse [59] embryos significantly increased glucose uptake when cultured under low O_2_ tension.

At implantation, VEGF plays an important role in endometrial vascular permeability by direct induction of angiogenesis, by recruiting endothelial cells, and by stimulating their proliferation [60, 61]. VEGF expression was higher in the 3% O_2_ group than in the 20% O_2_ group (*P* < 0.0001), suggesting that the ability for subsequent angiogenesis during implantation was increased in embryos cultured under hypoxic conditions. Increasing the expression of GLUT-3 and VEGF may contribute to promoting embryonic development during early preimplantation under hypoxic conditions. However, although GLUT-3 and VEGF genes are well known to be targets of HIF, we found that low O_2_ did not affect the protein level of HIF-1α (data not shown). Hence, we propose that HIF-2α, but not HIF-1α, plays an important role in gene regulation during hypoxia.

The successful implantation of embryos depends on steroid hormones, growth factors, and cytokine interactions between the specific receptors in the developing embryo and the endometrium via paracrine or autocrine pathways [62]. During the onset of the implantation window, leukemia inhibitory factor (LIF), a cytokine of the interleukin-6 superfamily that is maximally expressed by the endometrium, has a crucial role in preparing the embryo for implantation, through binding to its receptor complex, LIF receptors (LIFRs), and glycoprotein gp130 expressed in blastocysts [63, 64] to activate signaling transduction pathways between the uterus and embryo during the preimplantation period [65, 66]. According to our HIF-2α immunofluorescence results, these data suggest that the higher levels of LIFR in the blastocyst cultured in 3% O_2_ may enhance embryo attachment for subsequent implantation. Nevertheless, given the differences in implantation mechanism between mouse and human, the limitation of this study is based on mouse model and the applicability in human embryo requires further investigation. Combined with the VEGF findings reported here, this indicates that the mechanism involving hypoxia that modulates LIFR expression merits further investigation.

Mitochondrial membrane potential is a key indicator of mitochondrial activity, because it reflects the process of electron transport and oxidative phosphorylation, the driving force behind ATP production [23]. During mammalian embryogenesis, mitochondria were found to have altered patterns of both membrane potential and distribution, suggesting altered oocytes [67, 68].

Our JC-1 staining results showed that embryos in the 3% O_2_ group had increased MtMP. Our previous GLUT-3 expression results indicate that this higher MtMP may be the result of increased glucose intake by cells during hypoxia. Alternatively, decreased MtMP could lead to lower ATP generation, which could cause embryo development to be arrested or be abnormal because of energy insufficiency [69–71]. ROS are well known to induce apoptosis [72, 73], and O_2_ concentration is an exogenous factor that can enhance the generation of ROS by embryos [46]. When mouse [25] and bovine [36] embryos are cultured in atmospheric O_2_ (20%), there is a subsequent increase in the production of ROS, which have toxic effects on the embryo through the induction of apoptosis. We demonstrated that the number of apoptotic cells in blastocysts cultured under 20% O_2_ was significantly increased, probably associated with increased ROS, resulting in embryonic oxidative stress injury. These findings are consistent with those of Yoon et al. [36]. In contrast, embryos cultured in 3% O_2_ contain few apoptotic cells and undetectable DNA damage, explaining the better rate of development and hatching in embryos from the 3% O_2_ group.

Overall, the immunofluorescence staining and TUNEL assay results support our hypothesis that hypoxic conditions can increase protection against apoptosis and increase activity in the mitochondria to improve embryo development following in vitro culture. Future studies will be aimed at investigating ATP production within blastocysts developed under low O_2_ concentrations.

## Conclusion

**Fig. 5:**
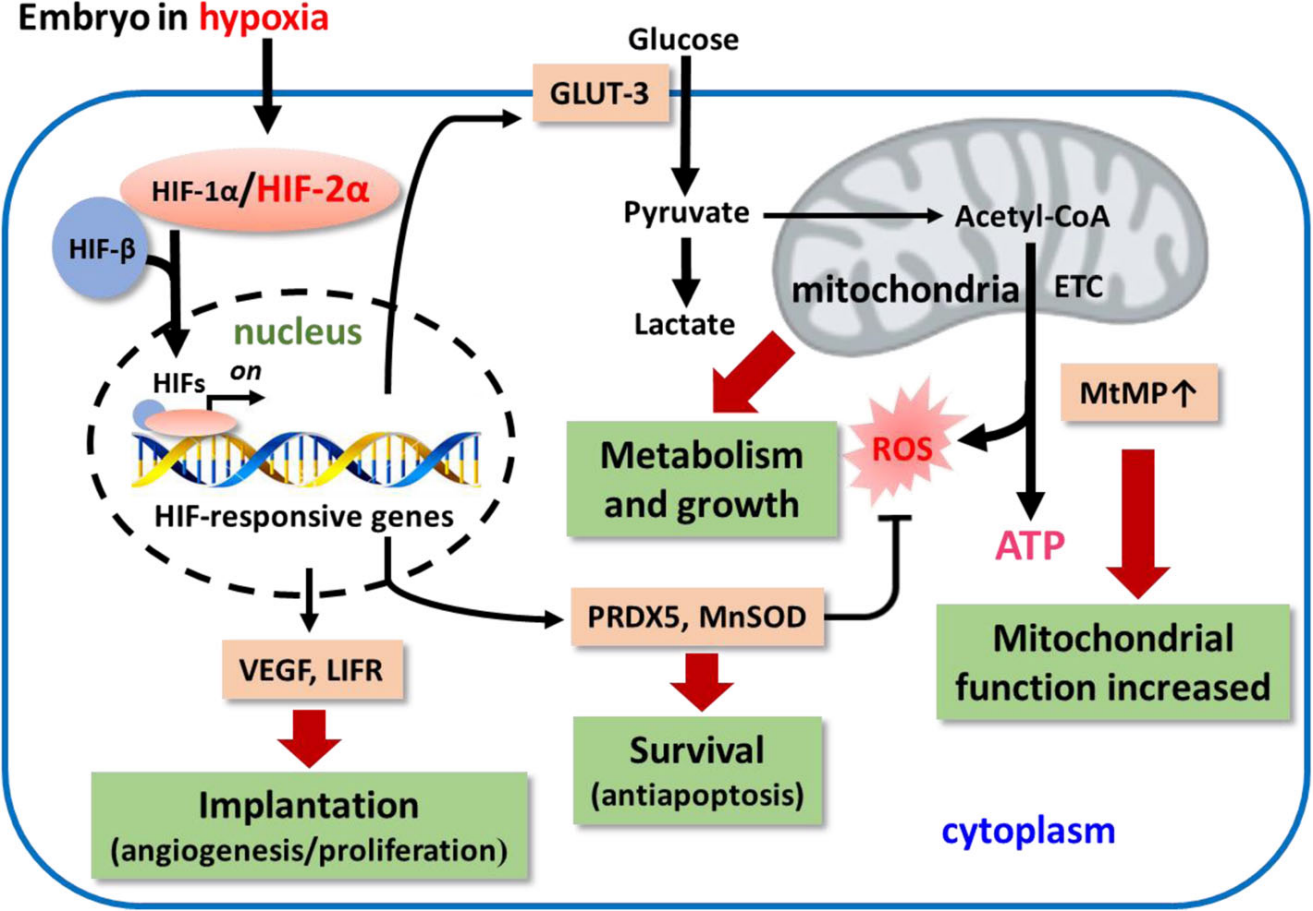
Hypothetical model for the role of HIF-2α in embryo development under hypoxia

These data confirm that preimplantation embryos have the ability to detect a lower O_2_ environment and respond to it by changes in expression of O_2_-regulated (HIF-2α) and antioxidant genes. Optimal oxygen tension is a critical factor in improving embryo competence during in vitro culture. In mouse model, the embryos have to encounter a decreasing oxygen gradient as they develop (from oviduct to the uterus). Whereas, Nastri et al., observed in low O_2_ tension only a small improvement (~5%) in live birth and clinical pregnancy rates, the evidence was of very low quality [74]. Hence we await further studies of suitable O_2_ tension in human embryos. This study indicates that low O_2_ tension provides a more conducive environment for embryo culture at least in mouse model. This environment increases antioxidant and mitochondrial function and promotes embryonic glycolysis, angiogenesis and antiapoptotic effect which could enhance implantation and hatching. These effects may be critically mediated by HIF-2α in hypoxic responses (Fig. 5).
